# Hydroxyl group's effects on the activity and durability of supported carbon–TiO_2_ for proton exchange membrane fuel cells

**DOI:** 10.1039/d5ra08718j

**Published:** 2026-02-24

**Authors:** Su-Jin Jang, Yi Kyeong Jung, Jeong Han Lee, Seok Hee Lee, Tae Ho Shin, Young Wook Lee

**Affiliations:** a Korea Institute of Ceramic Engineering & Technology Jin-ju 52851 Republic of Korea lsh@kicet.re.kr ths@kicet.re.kr; b Department of Education Chemistry and Research Institute of Advanced Chemistry Gyeongsang National University Jinju 52828 Korea lyw2020@gnu.ac.kr

## Abstract

Pt catalysts used for the cathode in proton exchange membrane fuel cells (PEMFCs) are mostly supported on carbon materials. However, durability issues arise under operating conditions due to carbon corrosion, which is a critical degradation mechanism. To improve the support durability, metal oxide supports combined with carbon have been extensively investigated. In this study, oxygen vacancy and hydroxyl group modified TiO_2_ particles supporting Pt nanoparticles were developed for applications in the oxygen reduction reaction (ORR) and the membrane electrode assemblies (MEAs). The Pt catalyst supports were prepared using a microwave-assisted method, and their performance was compared according to the degree of TiO_2_ crystallinity with and without heat treatment. X-ray diffraction (XRD) measurements before and after heat treatment revealed differences in the crystallinity of TiO_2_, showing the presence of anatase TiO_2_ and Ti–OH species. In the uncalcined carbon–TiO_2_ composite, the presence of titanium hydroxide (Ti–OH) species was confirmed by X-ray photoelectron spectroscopy (XPS) and attenuated total reflection (ATR) spectroscopy. The prepared Pt nanoparticle/TiO_2_–carbon (TiO_2_–Pt/SC) and Pt nanoparticle/heat-treated TiO_2_–carbon (TiO_2_–Pt/SC–H) catalysts exhibited superior ORR activity compared to that of the calcined catalyst. The effect of Pt loading on the ORR performance was also examined, revealing enhanced activity in the presence of anatase TiO_2_, which is attributed to its strong metal–support interactions (SMSIs). In addition, MEA tests confirmed that these samples exhibited high activity and improved stability. The enhanced oxygen reduction kinetics are ascribed to water dissociation and the formation of surface-adsorbed hydroxyl moieties. We believe that the results described herein provide important implications for the development of durable TiO_2_–carbon hybrid supports for MEA applications.

## Abstract 2

We developed Pt nanoparticle/TiO_2_–carbon (TiO_2_–Pt/SC) and Pt nanoparticle/heat-treated TiO_2_–carbon (TiO_2_–Pt/SC–H) catalysts, which demonstrated superior oxygen reduction reaction activity compared to conventional Pt/C. Membrane electrode assembly tests confirmed high activity and enhanced stability.

## Graphical abstract

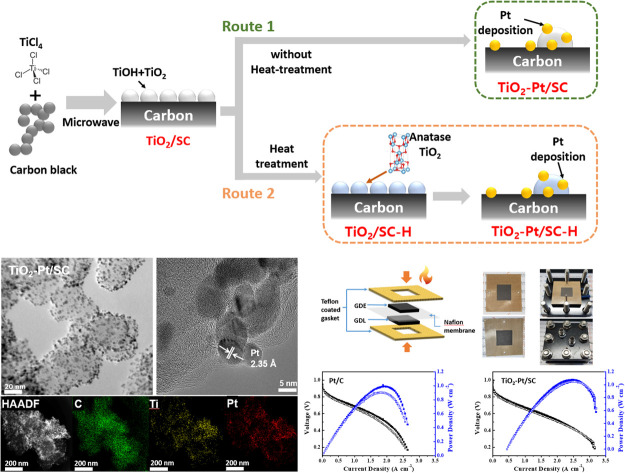

## Introduction

As environmental pollution caused by the extensive use of fossil fuels has become increasingly severe, research on green and sustainable energy technologies has intensified.^1^ Many countries and industries have made substantial investments in developing alternative and renewable energy sources capable of bridging the present and future energy demand-supply gap in a sustainable manner. These energy sources include solar, wind, hydropower, biomass, geothermal, and hydrogen energy, which encompasses fuel cell technologies. Among these, proton exchange membrane fuel cells (PEMFCs) have attracted significant attention due to their zero greenhouse gas emissions, high theoretical power density, and strong potential for sustainable energy systems.^2,3^ A PEMFC consists of a cathode, an anode, and a proton-conducting membrane, and extensive research has focused on catalyst and support materials to improve their electrocatalytic activity and stability. PEMFCs are particularly attractive for automotive applications, where the membrane electrode assembly (MEA), which generates electricity through electrochemical reactions, is the most critical component.^4^ Degradation of the MEA is a major cause for the performance loss of PEMFCs during long-term operations. Under PEMFC operating conditions, especially during startup and shutdown, the high electrode potential at cathodes can induce carbon oxidation and corrosion. This process leads to Pt dissolution, Pt particle agglomeration, and a significant decrease in the fuel cell performance and lifetime.^2,5–7^ As the degradation of Pt nanoparticles directly deteriorates the catalytic performance, improving the durability of the cathode catalyst is of critical importance. In particular, enhancing the binding energy between oxygen (O) or hydroxyl (OH) species and the catalyst surface has been suggested as an effective strategy.^8^ Moreover, improving the rate of the oxygen reduction reaction (ORR)—the rate-determining and slowest reaction in PEMFCs under all operating conditions—is essential.^3^ Loss of electrochemical surface area (ECSA), primarily caused by corrosion of the catalyst support and subsequent agglomeration of Pt nanoparticles, is widely recognized as a major challenge for catalyst durability.^9^ Numerous studies have reported that Pt dissolution and carbon support corrosion are responsible for the performance degradation of PEMFCs.^8–10^ Carbon materials are commonly used as catalyst supports due to their high electrical conductivity and large specific surface area.^6,11^ However, carbon corrosion and catalyst detachment severely impair fuel cell performance, highlighting the urgent need for corrosion-resistant support materials. To address this issue, hybrid catalyst supports composed of carbon and TiO_2_ have been proposed by tailoring the surface characteristics of carbon. Carbon corrosion at potentials around 0.9 V and Pt nanoparticle agglomeration lead to a decrease in ECSA and overall catalytic performance during fuel cell operation. Strong metal–support interaction (SMSI) between Pt and metal oxide supports has been shown to enhance the catalytic activity of Pt nanoparticles.^12,13^ Metal oxide supports can donate electrons to Pt nanoparticles, modifying the unfilled Pt d-band states and facilitating the adsorption, dissociation, and desorption of oxygen species. Among various metal oxides, titanium dioxide (TiO_2_) has been extensively investigated as a catalyst support for PEMFCs due to its high chemical stability and resistance to oxidation.^7,9^ The hypo-d-electron nature of TiO_2_ and related oxides (*e.g.*, ZrO_2_ and HfO_2_) enables strong metal–support interactions, resulting in enhanced nanoparticle dispersion and improved catalytic activity.^5,14,15^ TiO_2_ exists in several crystalline phases, including anatase, brookite, and rutile. In the absence of heat treatment, TiO_2_ typically exhibits low crystallinity or an amorphous structure. Depending on the synthesis method and precursor, TiO_2_ can possess varying oxidation states and surface functional groups. The spillover of surface hydroxyl species (M–OH) from metal oxides and their interactions play a critical role in electrocatalytic processes, particularly in ORR performance. Consumption of adsorbed M–OH species on the metallic catalyst surface reduces their chemical potential, thereby disturbing the established equilibrium and enhancing the catalytic activity.^14–16^ Furthermore, TiO_2_ containing oxygen vacancies and Ti^3+^ species has been reported to improve the ORR performance by lowering the kinetic energy barrier, modifying the OH adsorption energy on Pt surfaces, and altering the TiO_2_ band structure.^17^

In this study, we investigate the effect of TiO_2_ crystallinity in TiO_2_–carbon hybrid catalyst supports on stability and ORR activity, aiming to achieve performance comparable to that of conventional Pt/C catalysts. Low-crystallinity TiO_2_ and anatase TiO_2_ were deposited on carbon supports using a microwave-assisted synthesis method. Pt nanoparticles were uniformly dispersed on the prepared supports. The amorphous TiO_2_–Pt catalyst exhibited superior ORR activity, stability, and durability, as well as excellent MEA performance. The presence of oxygen vacancies and surface hydroxyl groups (OH) on TiO_2_ was found to play a crucial role in enhancing the electrochemical performance.

## Experimental

### Materials

SP carbon was obtained from VINATech Co., Ltd and titanium(iv) tetrachloride (TiCl_4_, 99%, Wako Chemicals, Japan), ethylene glycol (99.8%, Sigma-Aldrich), chloroplatinic acid hexahydrate (H_2_PtCl_6_·6H_2_O, Sigma-Aldrich), isopropyl alcohol (IPA, 99.5%, Sigma-Aldrich), sodium hydroxide (NaOH, 97%, Sigma-Aldrich), and perchloric acid (HClO_4_, 70%, Sigma-Aldrich) were purchased. All the chemicals were used as received without further purification.

### Synthesis of the carbon-TiO_2_ composite

The SP-TiO_2_ composites were obtained by a microwave-assisted hydrothermal reaction without any surfactants. 0.1 g SP carbon was dispersed in 40 mL deionized water in a Teflon beaker. 2 M TiCl_4_ (in deionized water) was added to 0.4 mL under vigorous stirring. The microwave system was operated at 200 W, and the sample temperature was kept at 80 °C for 30 min. After the microwave process, the resultant slurry was filtered, washed with deionized water and ethanol, and dried in a drying oven at 80 °C. This product is denoted as TiO_2_/SC. To increase the crystallinity of TiO_2_, TiO_2_/SC was heat-treated at 350 °C in N_2_ atmosphere, and this product is denoted as TiO_2_/SC–H.

### Synthesis of TiO_2_–Pt/SC and TiO_2_–Pt/SC–H

The synthesis method of TiO_2_/SC and TiO_2_/SC–H (60 mg) was dispersed in 150 mL of ethylene glycol and sonicated for 10 min, respectively. 84 mg of H_2_PtCl_6_ was added to this solution and vigorously stirred for 10 min. The pH of the solution was adjusted to 12 by adding 5 M NaOH, and the mixed solution was continuously stirred for 60 min. The suspension was heated at 160 °C for 3 h. Thereafter, the suspension was filtered and washed with ethanol and distilled water. The obtained TiO_2_–Pt/SC and TiO_2_–Pt/SC–H were dried overnight at 80 °C.

### Characterization

Phase analysis was performed using powder X-ray diffraction (XRD) (Bruker Inc., D8-Advanced, Germany) with Cu Kα (*λ* = 1.5406 Å) radiation. X-ray photoelectron spectroscopy (XPS) was performed using a Thermo VG Scientific Sigma Probe spectrometer using an Al Kα X-ray (*λ* = 1486.6 eV) as the excitation source. The XPS data were calibrated using the C 1s peak at 284.5 eV. The morphologies of the samples were observed using transmission electron microscopy (TEM) (JEOL, JEM-4010, Japan). The oxygen and hydroxyls (OHs) were investigated by Fourier transform infrared-attenuated total reflectance (FTIR-ATR) (PerkinElmer Frontier, FT-IR/FIR Spectrometer, USA). Spectra were collected at room temperature over the range of 4000–500 cm^−1^ (16 scans, 4 cm^−1^ resolution) using the same FT-IR/FIR spectrophotometer as previously cited (PerkinElmer Frontier, Waltham, MA, USA), equipped with a germanium crystal.

### Electrochemical performance

Cyclic voltammetry (CV) and oxygen reduction reaction (ORR) activity measurements were performed in a three-electrode cell system using a VSP potentiostat (Biologic, France) and RRDE-3A rotating ring disk electrode model. The electrocatalytic activity of the hybrid samples was evaluated using a three-electrode setup with the counter, reference and working electrodes of Pt wire, Ag/AgCl, and glassy carbon (5 mm diameter), respectively. The catalyst solution was prepared by mixing the catalyst (10.0 mg), distilled water (280 µL), isopropyl alcohol (200 µL), and a 5 wt% Nafion solution (20 µL) under sonication for 1 h. Then, the catalyst (3 µL) was deposited on the glassy carbon electrode, and the working electrode was dried. The measured potential *vs.* Ag/AgCl was converted to the reversible hydrogen electrode (RHE) scale according to the Nernst equation:
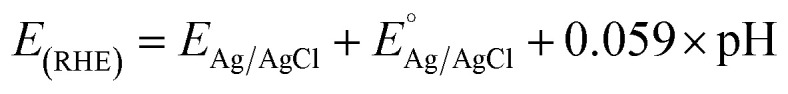
Here, *E*_RHE_ is the converted potential *vs.* RHE, 
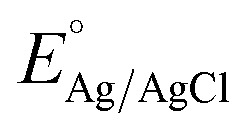
 is 0.1976 V at 25 °C, and *E*_Ag/AgCl_ is the experimentally measured potential *vs.* Ag/AgCl reference. The prepared working electrode was washed with highly purified water, and then electrochemically cleaned by multiple potential cycles between 0.05 and 1.10 V *vs.* RHE in 0.1 M HClO_4_ at a scan rate of 50 mV s^−1^. CV data were measured by sweeping the potential between 0.05 and 1.10 V *vs.* RHE at a scan rate of 20 mV s^−1^. The ORR polarization curves of the catalysts loaded on rotating disk electrodes (diameter = 5 mm) were obtained using the linear sweep voltammetry technique in O_2_-saturated 0.1 M HClO_4_ solution at a scan rate of 10 mV s^−1^ with a rotating speed of 1600 rpm. Furthermore, to study the durability of the catalysts, an accelerated stability test was conducted by cycling the working electrode between 0.6 and 1.0 V *vs.* RHE at 500 mV s^−1^ in a N_2_-saturated electrolyte for 5000 cycles. The ORR activity measurement and CV were taken every 1000 cycles to observe the stability area. The ECSA of Pt can be calculated from the coulombic charges accumulated during hydrogen adsorption or desorption after correcting for the double-layer charging current from the CVs as follows:
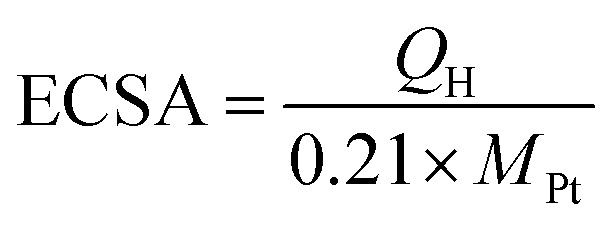
Here, *Q*_H_ (mC) is the charge due to the hydrogen adsorption/desorption in the hydrogen region of the CV, and 0.21 mC cm^−2^ is the electrical charge associated with the monolayer adsorption of hydrogen on Pt. *M*_Pt_ is the mass of Pt loaded on the working electrode.

### MEA performance

A commercially available gas diffusion layer (GDL, SGL-39BC, CNL) was used as the anode for the fuel cell experiments. The single cell was assembled by a commercial MEA (5 cm^2^ active area), GDL (SGL-39BC, CNL), Teflon gaskets, and Nafion 211 membrane, and operated for PEMFC. Catalyst inks were prepared by blending the sample with a Nafion (5 wt%) ionomer solution and isopropyl alcohol for 4 h. The catalyst ink was deposited on GDL with the Pt loading of 0.5 mg cm^−2^ on the cathode and the anode loading was 0.5 mg cm^−2^. Then, the single cell was installed at a fuel cell station (CNL Energy). The cell operation temperature was 70 °C and the relative humidity was 100%, while the stoichiometry of hydrogen and O_2_ was 105 and 330 cm^3^ min^−1^, respectively.

## Results & discussion

**Scheme 1:**
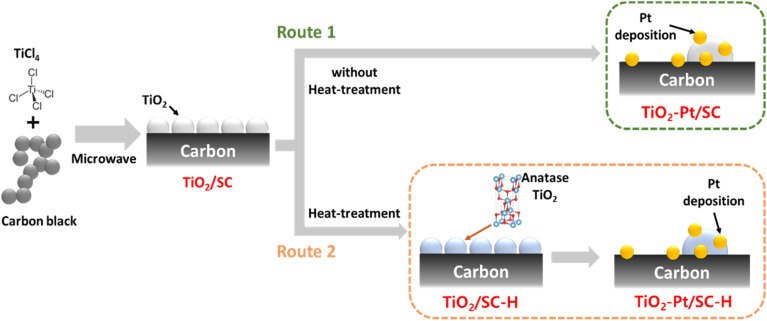
Synthesis method of TiO_2_–Pt/SC and TiO_2_–Pt/SC–H.

Scheme 1 schematically depicts the synthesis of the Pt catalysts for PEMFC. We supported the Pt catalysts on TiO_2_/SC *via* the microwave-assisted process. In this system, TiO_2_/SC has low crystallinity and mixed hydroxyl groups with anatase. After heat treatment at 350 °C, TiO_2_ has an anatase structure verifying oxygen content named as TiO_2_–Pt/SC–H. The support obtained by the above method was compared to that without additional heat treatment. As route 2, TiO_2_–SC was heated under nitrogen at 350 °C to obtain the anatase TiO_2_. The catalysts supported on the sample by routes 1 and 2 were synthesized *via* ethylene glycol reduction of the Pt precursor at 160 °C.

**Fig. 1:**
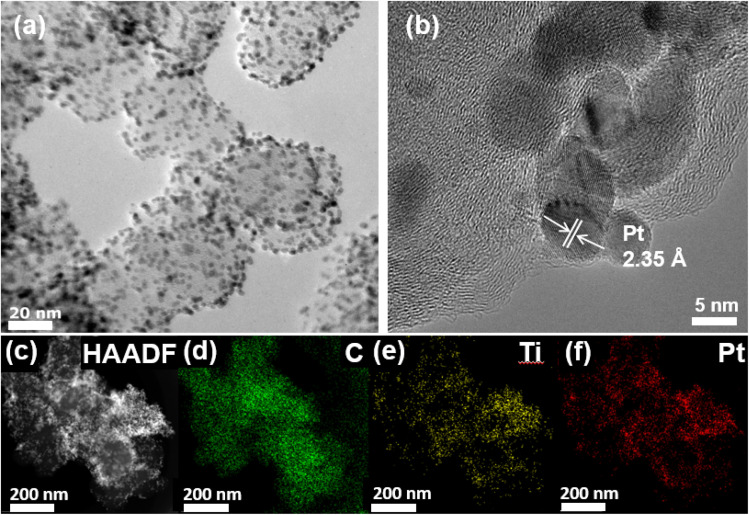
(a) TEM image of TiO_2_–Pt/SC. (b) HR-TEM image of a Pt particle on TiO_2_–carbon. (c) HADDF-STEM image and (d–f) corresponding EDS elemental mapping images of TiO_2_–Pt/SC.

As shown in Fig. 1a, the structure and morphology of TiO_2_–Pt/SC were investigated by transmission electron microscopy (TEM), revealing that the Pt nanoparticles (NPs) are homogeneously dispersed on the TiO_2_–carbon surface. In the corresponding high-resolution TEM (HR-TEM) images, lattice fringes with a spacing of 2.32 Å were observed, which can be assigned to the (111) crystal plane of Pt nanoparticles in TiO_2_–Pt/SC.^18,19^ The Pt nanoparticles supported on TiO_2_–Pt/SC exhibit a smaller particle size, which can be attributed to the more oxidized TiO_2_ surface being electron-deficient and the resulting lower diffusion rates of the Pt species.^20–22^ In contrast, the lattice fringes of TiO_2_ are not clearly observed in the HR-TEM images, likely due to its amorphous structure and disordered lattice, as shown in Fig. 1b.^23^ The average particle size of the Pt nanoparticles was determined to be 3.5 ± 1.4 nm (Fig. 1b). The compositional structure of TiO_2_–Pt/SC was further confirmed by elemental mapping using high-angle annular dark-field scanning transmission electron microscopy (HAADF-STEM) coupled with energy-dispersive X-ray spectroscopy (EDS), which clearly reveals the spatial distribution of Pt (red), C (green), and Ti (yellow), as shown in Fig. 1c–f. These results indicate that Pt nanoparticles are uniformly dispersed on the surfaces of both TiO_2_ and SP carbon, even though TiO_2_ partially covers the carbon surface (Fig. 1). For the TiO_2_–Pt/SC–H sample synthesized after heat treatment, the formation of TiO_2_ was confirmed by the observed lattice spacing and EDS elemental mapping obtained from TEM analysis. The average particle size of the Pt nanoparticles in TiO_2_–Pt/SC–H was determined to be 3.9 ± 1.3 nm (Fig. S1). In general, when TiO_2_ is synthesized in the presence of a metal species, hydroxyl (OH^−^) groups are removed.^24^ Accordingly, the formation mechanism of the synthesized catalysts can be described as follows: TiO_2_–Pt/SC and TiO_2_/SC–H are generated as a result of the removal of the surface OH groups induced by metal incorporation. These bands are associated with bridging OH species adsorbed on Ti atoms with different valence states. The hydroxyl species present on the TiO_2_ additive are likely to interact with interfacial water molecules through hydrogen bonding, inducing structural rearrangements in the interfacial water layer. Such structural changes effectively increase the local concentration of OH species near the electrode surface, thereby enhancing the supply of hydroxyl species to the Pt surface.^25,26^ The crystal structure of the TiO_2_–carbon composite was investigated by X-ray diffraction (XRD), which reveals the presence of both TiO_2_ and carbon phases. A diffraction peak observed at approximately 25° can be indexed to the (002) plane of carbon. In addition, the diffraction peaks of TiO_2_/SC and TiO_2_/SC–H located at 2*θ* values of 25.38°, 32.00°, and 38.11° correspond to the (101), (110), and (200) planes of anatase TiO_2_, respectively (JCPDS no. 21-1276).^27,28^ The broad diffraction feature observed in the 30°–40° range for TiO_2_/SC is attributed to the presence of the titanium hydroxide (Ti–OH) species (Fig. 1a).^29^ The weak crystallinity of TiO_2_ in TiO_2_/SC is consistent with the amorphous characteristics observed in the TEM images. As shown in Fig. S2a, diffraction peaks at 39.8°, 46.89°, and 68.38° can be assigned to the (111), (200), and (220) planes of the Pt nanoparticles, respectively (JCPDS no. 04-0802).^30^ After heat treatment, the crystallinity of TiO_2_ was significantly enhanced, and the formation of anatase TiO_2_ was further confirmed by lattice spacing measurements obtained from both XRD analysis and TEM images (Fig. S2a and b).

**Fig. 2:**
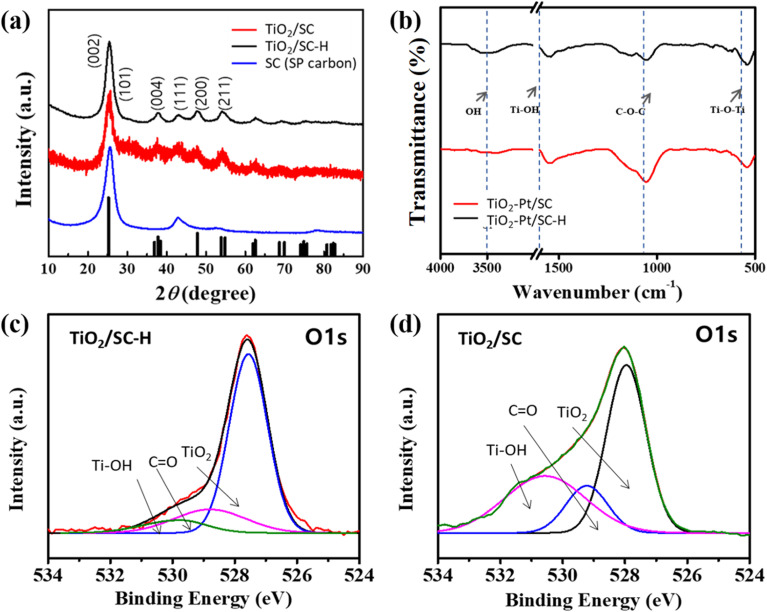
(a) XRD patterns of SC, TiO_2_/SC and TiO_2_/SC–H. (b) ATR spectra of TiO_2_/SC and TiO_2_/SC–H on the Pt–support. XPS spectra with O 1s of (c) TiO_2_/SC–H and (d) TiO_2_/SC.

Attenuated total reflectance (ATR) spectroscopy was performed to further investigate the oxygen species and surface hydroxyl groups on TiO_2_. The ATR spectra of Pt-loaded TiO_2_/SC and TiO_2_/SC–H were collected in the range of 500–4000 cm^−1^. The broad absorption band observed at 3400–3600 cm^−1^ is attributed to weakly bound surface hydroxyl groups with lateral interactions on TiO_2_–Pt/SC, and the broadening of this band indicates increased surface oxidation. The absorption band centered at approximately 3150 cm^−1^ is assigned to ordered hydroxyl (OH) species on the Ti surface (Fig. 2b).^30,31^ To further examine the surface oxygen states, X-ray photoelectron spectroscopy (XPS) was conducted to analyze the surface composition and chemical states of TiO_2_–Pt/SC and TiO_2_–Pt/SC–H. As shown in Fig. 2c and d, the O 1s spectra can be deconvoluted into three components: a peak at 531.0 eV corresponding to oxygen vacancies (V_0_) and surface hydroxyl groups (–OH), a peak at 529.5 eV assigned to lattice oxygen (O^2−^) in the Ti–O bonds of TiO_2_, and a peak at 527.8 eV attributed to weakly bound Ti–O species on the TiO_2_ surface.^19,30^ To elucidate the effect of carbon, additional measurements were performed on bulk TiO_2_ and heat-treated TiO_2_, and similar trends in the oxygen-related peaks were observed (Fig. S3). Overall, heat treatment induces the reoxidation of TiO_2_, leading to improved crystallinity and a reduction in the intensity of oxygen vacancy- and hydroxyl-related peaks. To investigate the influence of the oxygen-related surface species on the surface catalytic activity, the electrocatalytic performance of the synthesized catalysts was evaluated. Platinum nanoparticles are widely employed as catalysts for the oxygen reduction reaction (ORR) in fuel cells, and extensive efforts have been devoted to improving their catalytic activity and stability. In this study, the synthesized catalyst was applied to the ORR to evaluate its electrochemical performance. The ORR in fuel cells is strongly dependent on shifts in the d-band center of the catalyst. Strong metal–support interactions (SMSI) between TiO_2_ and Pt induce electron transfer from the support to Pt, leading to a downward shift of the Pt d-band center and, consequently, enhanced catalytic activity. This mechanism has been reported in theoretical studies of the ORR on Pt/C and TiO_2_–Pt systems, and such interactions are also known to improve catalyst durability.^31,32^

**Fig. 3:**
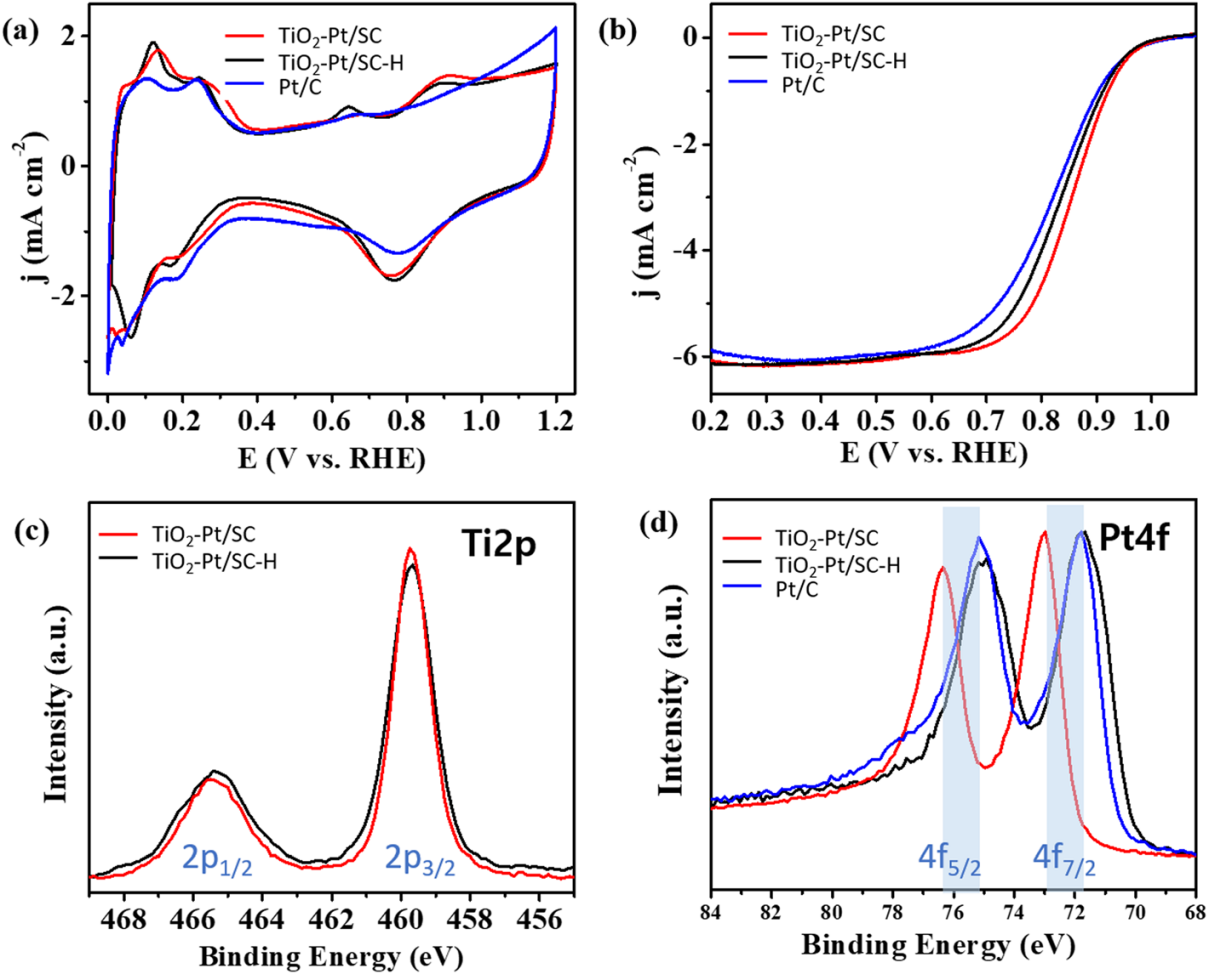
(a) CVs of TiO_2_–Pt/SC, TiO_2_–Pt/SC–H, and Pt/C for 0.1 M HClO_4_ at a scan rate of 50 mV s^−1^. (b) ORR curves of TiO_2_–Pt/SC, TiO_2_–Pt/SC–H, and Pt/C in O_2_-saturated 0.1 M HClO_4_ at a scan rate of 10 mV s^−1^. Ti 2p (c) and Pt 4f (d) XPS spectra of TiO_2_–Pt/SC and TiO_2_–Pt/SC–H.

**Fig. 4:**
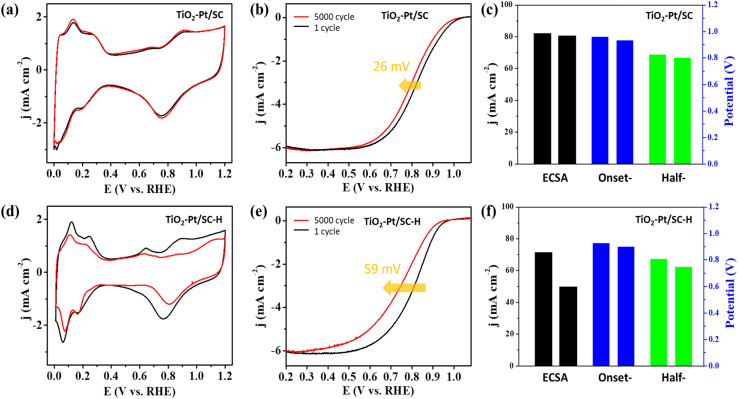
(a and d) CV curves and (b and e) ORR curves of TiO_2_–Pt/SC and TiO_2_–Pt/SC–H for the durability test over 5000 cycles, respectively. (c and f) ECSA, onset-potential, and half-potential of TiO_2_–Pt/SC and TiO_2_–Pt/SC–H, respectively.

Fig. 3a presents the cyclic voltammograms (CVs) of TiO_2_–Pt/SC and TiO_2_–Pt/SC–H recorded in a 0.1 M HClO_4_ electrolyte, which were used to determine the electrochemically active surface areas (ECSAs) of the corresponding catalysts. The influence of TiO_2_ on the electrocatalytic performance was evaluated through electrochemical measurements. The specific ECSAs, normalized to the Pt mass, were calculated by integrating the charge associated with the hydrogen adsorption/desorption region after correction for the double-layer contribution. The ECSAs of TiO_2_–Pt/SC, TiO_2_–Pt/SC–H, and Pt/C were determined to be 82.1, 71.3, and 66.8 m^2^ g^−1^, respectively. To evaluate the catalytic activity toward the oxygen reduction reaction (ORR), polarization curves were recorded in O_2_-saturated 0.1 M HClO_4_ solution at a scan rate of 10 mV s^−1^ and a rotation speed of 1600 rpm (Fig. 3b). A comparison of the ORR performance of TiO_2_–Pt/SC–H, TiO_2_–Pt/SC, and Pt/C revealed that TiO_2_–Pt/SC exhibited superior catalytic activity. In particular, the current density of TiO_2_–Pt/SC was approximately 0.2 mA cm^−2^ higher than that of TiO_2_–Pt/SC–H at 0.9 V, indicating a significant enhancement in the ORR electrocatalytic activity (Fig. 3b). This improvement can be attributed to the presence of TiO_2_ on the catalyst surface, which facilitates the dissociation of oxygen and water molecules and enhances oxygen adsorption on the support. The adsorbed oxygen species can subsequently migrate to adjacent Pt active sites *via* surface diffusion, thereby promoting the ORR.^33,34^ In addition, the enhanced ORR performance of TiO_2_–Pt/SC and TiO_2_–Pt/SC–H can be attributed to strong metal–support interactions (SMSI) between the Pt nanoparticles and the TiO_2_-containing support. These strong interactions lead to tight interfacial bonding, which effectively suppresses Pt migration and aggregation, thereby improving both catalytic stability and activity. The SMSI effect is likely associated with the relatively electron-rich nature of the Pt nanoparticles formed on the TiO_2_ support, enabling electron donation from Pt to the support *via* the Pt d-band centers. This electronic interaction weakens the Pt–O bond strength, which in turn facilitates ORR kinetics. As a result, TiO_2_–Pt/SC exhibited the highest ORR activity, outperforming previously reported catalysts (Table S1). Since electrochemical reactions of the catalysts primarily occur at the surface, X-ray photoelectron spectroscopy (XPS) was employed to analyze the surface binding energies of the metal species. The Ti 2p XPS spectrum exhibits characteristic doublet peaks at approximately 459.4 and 465.1 eV, corresponding to the Ti 2p_3_/_2_ and Ti 2p_1_/_2_ states, respectively (Fig. 3c). The spin–orbit splitting of 5.7 eV confirms the presence of Ti exclusively in the Ti^4+^ oxidation state. Compared with pristine TiO_2_, the Ti 2p peaks show a slight shift toward higher binding energies, suggesting a modification of the Ti chemical environment, likely arising from strong interactions with the carbon support. In contrast, the broader Ti 2p peaks observed for titanium powder are attributed to the coexistence of metallic Ti and sub-stoichiometric oxides associated with lower oxidation states (Ti^2+^ and Ti^3+^), which are present beneath the anatase layer covering the powder surface. As shown in Fig. 3d, the Pt 4f XPS spectra display asymmetric 4f_7_/_2_ and 4f_5_/_2_ spin–orbit components, a characteristic feature of metallic Pt (Pt^0^). The presence of higher binding energy components indicates a partial oxidation state of Pt.^35^ After heat treatment, an increase in the Pt binding energy is observed, which can be attributed to electron transfer from Pt to the oxide support. Furthermore, the noticeable positive shift of approximately 1.2 eV relative to TiO_2_–Pt/SC–H is ascribed to enhanced metal–support interactions. Analysis of the valence band XPS spectra reveals that the d-band center positions of TiO_2_–Pt/SC and TiO_2_–Pt/SC–H are located at −4.41 and −4.04 eV, respectively (Fig. S4). The downshift of the Pt 5d-band center is known to enhance the ORR activity by lowering the energy level of the antibonding orbitals associated with oxygen adsorption on Pt (Pt–O) relative to the Fermi level, thereby weakening the Pt–O bond strength.^33^ The metal–support interactions reduce the adsorption strength of oxygen-containing intermediates formed on the Pt surface during the rate-determining step of the oxygen reduction reaction (ORR), thereby accelerating the overall ORR kinetics. In addition, the reaction between the surface-bound Pt–OH species and oxygen constitutes a critical step in the ORR pathway, which is consistent with the enhanced ORR performance observed. The Pt–TiO_2_ nanocomposites exhibit superior electrocatalytic activity toward oxygen reduction, which can be attributed to the hypo-d-electron character of titanium oxide. This electronic feature facilitates the effective spillover of the adsorbed hydroxyl species from the hypo-d-electron oxide support to the Pt surface at the Pt–TiO_2_ interface. Since hypo-d-electron oxides possess ion-exchange membrane-like properties, a higher valence capacity promotes a more pronounced spillover effect.^15^ Furthermore, the enhanced electrocatalytic activity of the Pt catalyst is associated with the formation of crystalline TiO_2_. The stability of the catalysts was evaluated through repeated cyclic voltammetry (CV) measurements. Catalyst durability was further examined by conducting accelerated degradation tests (ADTs) *via* CV in the potential range of 1.0–1.5 V at a scan rate of 500 mV s^−1^. Fig. 4 presents the ORR polarization curves and CV profiles recorded before and after the durability tests. Both catalysts exhibited a decrease in the electrochemically active surface area (ECSA) after maintaining the potential at 1.23 V for 1 h, which is attributed to the Pt migration and particle growth during prolonged cycling. Notably, after 5000 cycles, the ECSA loss of TiO_2_–Pt/SC (Fig. 4a) was significantly smaller than that of TiO_2_–Pt/SC–H (Fig. 4d), demonstrating its superior durability.

The electrochemical activity of the TiO_2_–Pt/SC catalyst is slightly higher than that of the commercial Pt/C catalyst, which can be attributed to the similar electronic structures of Ti and Pt, as both elements belong to the d-block and possess comparable outer electronic configurations. The interaction between Ti and Pt is facilitated by the overlap between the occupied d orbitals of Pt and the vacant d orbitals of Ti, promoting strong interfacial interactions. These interactions effectively anchor the Pt nanoparticles, suppressing their migration and agglomeration. Moreover, the electronic interaction between TiO_2_ and Pt modifies the electronic structure of Pt, thereby enhancing the intrinsic catalytic activity.

**Fig. 5:**
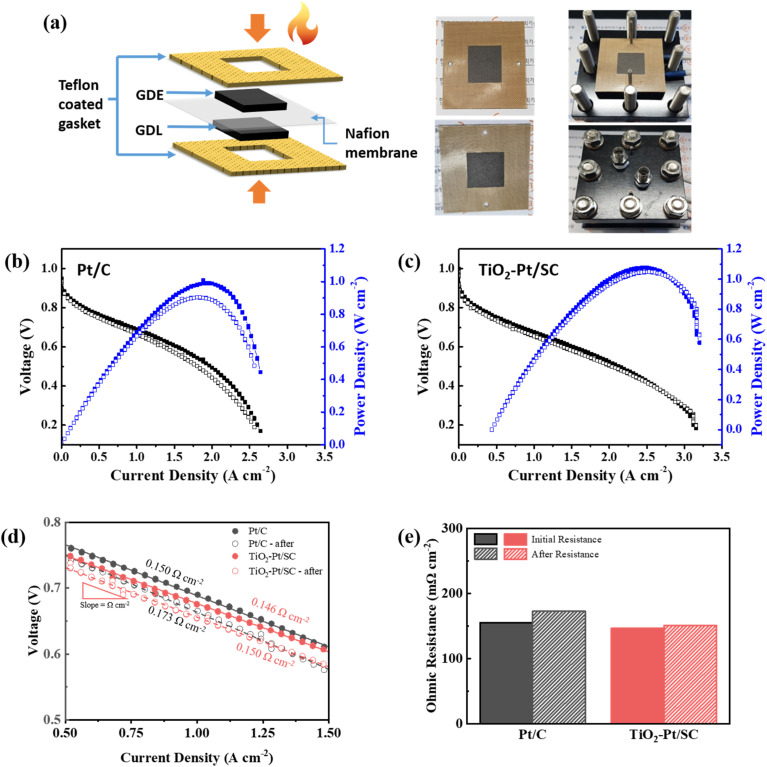
(a) Schematic of the preparation of 5 cm^2^ MEA. (b and c) *I*–*V* polarization and power density curves of TiO_2_–Pt/SC and commercial Pt/C. (d) Linear part of the *I*–*V* curves in part used to determine the ohmic resistance and (e) ohmic resistance before and after AST.

The prepared TiO_2_–Pt/SC catalyst and commercial Pt/C were employed as cathode catalysts in PEMFCs, as schematically illustrated in Fig. 5a, with a catalyst loading of 0.5 mg cm^−2^. Fig. 5b and c present the current density–voltage (*I*–*V*) polarization curves of the membrane electrode assemblies (MEAs) recorded in an H_2_/O_2_ atmosphere before and after the accelerated stress test (AST). The effect of potential cycling on the overall cell performance was minimal. Nafion® 212 was used as the proton exchange membrane, and the effective electrode area was 5 cm^2^. Both gas humidification and cell operation were conducted at 70 °C. The AST was performed by holding the cell at 1.23 V for 24 h. After the AST, the open-circuit voltage (OCV) slightly decreased from 1.00 to 0.978 V. The polarization curves can be divided into three distinct regions: activation polarization, ohmic polarization, and concentration polarization. For the TiO_2_–Pt/SC catalyst, current densities of 237 mA cm^−2^ at 0.80 V and 1519 mA cm^−2^ at 0.60 V were achieved, whereas the Pt/C catalyst delivered 282 mA cm^−2^ at 0.80 V and 1522 mA cm^−2^ at 0.60 V. The maximum power densities (MPDs) of TiO_2_–Pt/SC and Pt/C were measured to be 1076 and 1050 mW cm^−2^, respectively. In the low current density region, the Pt/C catalyst exhibited higher electrochemical polarization, indicating slower electrochemical reaction kinetics. This behavior may be attributed to differences in the electrode wettability during the initial discharge stage. The faster electrode wettability of the Pt/C catalyst facilitates proton conduction, thereby reducing electrochemical polarization. The superior stability of TiO_2_–Pt/SC is attributed to strong metal–support interactions (SMSI) between the Pt nanoparticles and the carbon/TiO_2_ support, which effectively suppress Pt detachment and subsequent agglomeration.^19,35^ As shown in Fig. 5d, the ohmic resistance was calculated from the slope of the *I*–*V* curve within the linear range from 0.5 to 1.0 A cm^−2^; the value of TiO_2_–Pt/SC (0.146 Ω cm^2^) was similar to that of Pt/C (0.150 Ω cm^2^) before cycling. This similarity might imply that the carbon/TiO_2_ support is sufficiently conductive to be used as a catalyst support, comparable to the commercial carbon black in Pt/C. Therefore, we could infer that the poor electrical conductivity issues of the ceramic support could be mitigated by the carbon/TiO_2_ assembly. Furthermore, the ohmic resistance of TiO_2_–Pt/SC increased by only 2.74% after AST, which is a very small change, indicating the robustness of the TiO_2_/SC support. On the other hand, the resistance of Pt/C increased drastically from 0.150 to 0.173 Ω cm^2^, which is an increase of 15.3% (Fig. 5d and e). The stability of the synthesized TiO_2_–Pt/SC and Pd/C was evaluated through repeated cyclic voltammetry (CV) measurements.^36,37^ As a result, the PEMFC employing TiO_2_–Pt/SC as the cathode catalyst exhibited better performance than that using the commercial Pt/C catalyst. The current density and decay rate values measured before and after accelerated stress tests (ASTs) are summarized in Table S2 and Fig. S5. Notably, the TiO_2_–Pt/SC-based fuel cell maintained a high current density and stable cyclic voltammetry (CV) characteristics after accelerated durability testing.

## Conclusion

In this work, a TiO_2_–carbon hybrid support for Pt catalysts was successfully synthesized *via* a simple microwave-assisted process. The crystallinity and phase composition of TiO_2_ were found to vary, depending on the presence or absence of post-synthesis heat treatment. In the absence of a highly crystalline anatase TiO_2_ matrix, TiO_2_–Pt/SC exhibited higher ORR activity than TiO_2_–Pt/SC–H, which can be attributed to the presence of surface hydroxyl (–OH) groups and their associated binding energies. Electrocatalytic ORR activity and durability evaluations demonstrated that TiO_2_–Pt/SC possesses superior resistance to carbon corrosion compared with TiO_2_–Pt/SC–H, owing to strong metal–support interactions (SMSI). After accelerated stress tests (ASTs), the electrochemically active surface area (ECSA) loss of TiO_2_–Pt/SC was limited to only 6.41%, whereas the commercial Pt/C catalyst exhibited a significantly higher loss of 29.86%. Furthermore, membrane electrode assembly (MEA) tests conducted under AST protocols revealed that TiO_2_–Pt/SC maintained excellent fuel cell performance, showing only a 2.42% decrease in maximum power density after holding at 1.23 V. Overall, this study provides a feasible strategy for the synthesis of carbon–metal oxide hybrid composite supports for Pt catalysts. Moreover, optimization of surface functional groups on the support offers a promising pathway for the development of high-performance cathode catalysts for proton exchange membrane fuel cells (PEMFCs).

## Conflicts of interest

There are no conflicts to declare.

## Supplementary Material

RA-016-D5RA08718J-s001

## Data Availability

The supplementary information (SI) file includes all data supporting the article. Supplementary information is available. See DOI: https://doi.org/10.1039/d5ra08718j.
