# Supplementary material for: Hydroxyl group's effects on the activity and durability of supported carbon–TiO2 for proton exchange membrane fuel cells
Source: RSC Adv. 2026 Feb 24;16(12):10925–34. doi: 10.1039/d5ra08718j (PMC12930248; doi:10.1039/d5ra08718j)
Supplement: RA-016-D5RA08718J-s001 [file RA-016-D5RA08718J-s001.pdf]

Supplementary Information (SI) for RSC Advances.
This journal is © The Royal Society of Chemistry 2026

Supporting Information for

Hydroxyl Group on the Activity and Durability of Supported Carbon-TiO_2 for the Proton Exchange Membrane Fuel Cells

Su-Jin Jang[a], Yi Kyeong Jung[a], Jeong Han Lee[a], Seoyoon Shin[a] , Seok Hee Lee[a], Tae Ho Shin[a*] and Young Wook Lee[b*]*

[a]Korea Institute of Ceramic Engineering & Technology, Jin-ju 52851, Republic of Korea

[b]Department of Education Chemistry and Research Institute of Advanced Chemistry Gyeongsang National University, Jinju 52828, Korea

*Corresponding author. *Seok Hee Lee, Tae Ho Shin, Young Wook Lee*
E-mail: lsh@kicet.re.kr, ths@kicet.re.kr, lyw2020@gnu.ac.kr

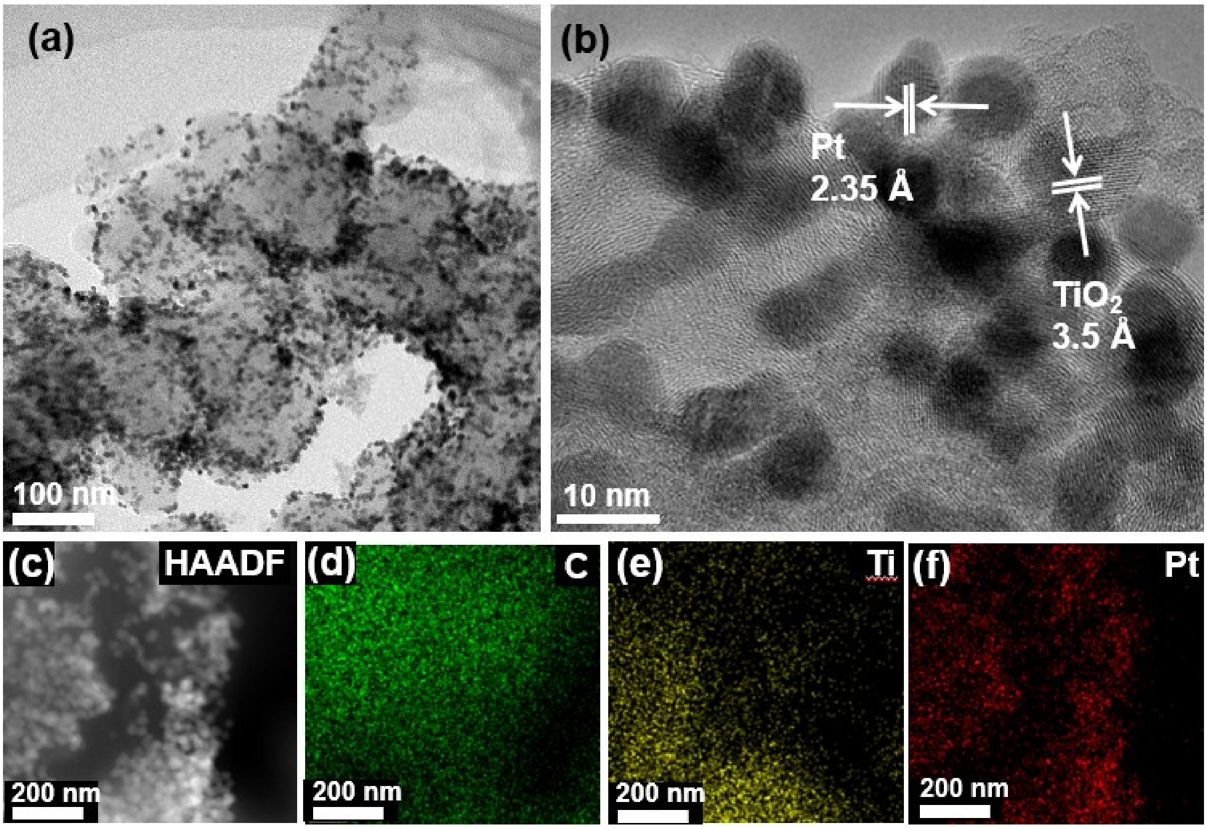

Figure S1. (a) TEM image of TiO_2-Pt/SC-H. (b) HRTEM image of a Pt particle on TiO_2-carbon. (c) HADDF-STEM image and (d-f) corresponding EDS elemental mapping images of TiO_2-Pt/SC-H.

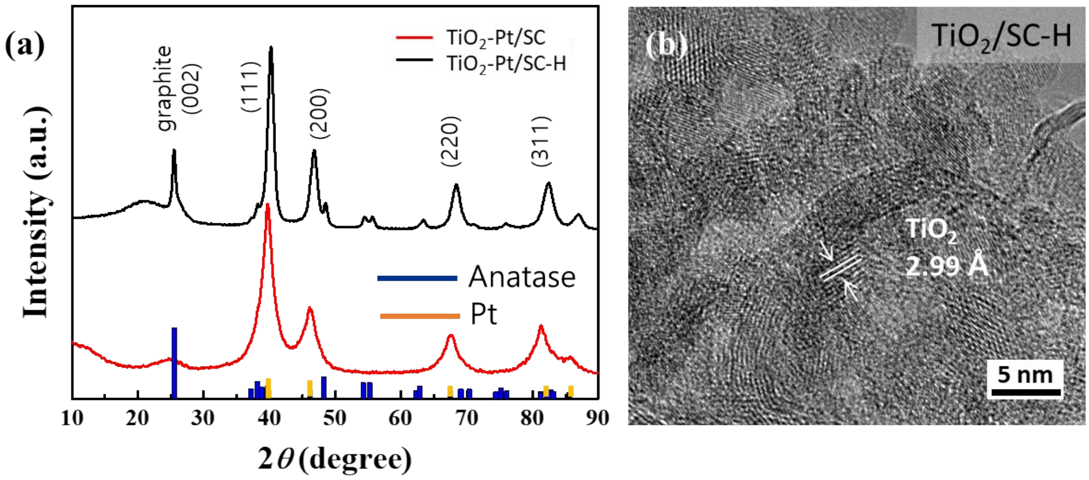

Figure S2. (a) XRD patterns and (b) TEM of TiO_2-Pt/SC-H TEM images.

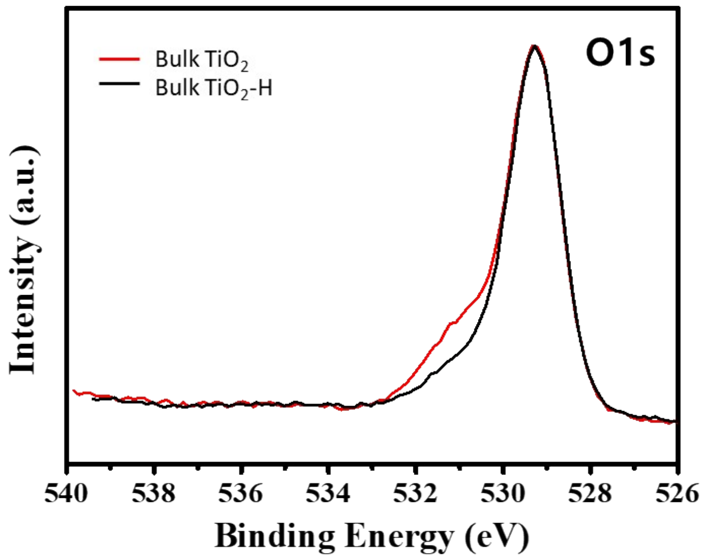

Figure S3. XPS profiles in O1s region for bulk TiO_2 and anatase TiO_2-H.

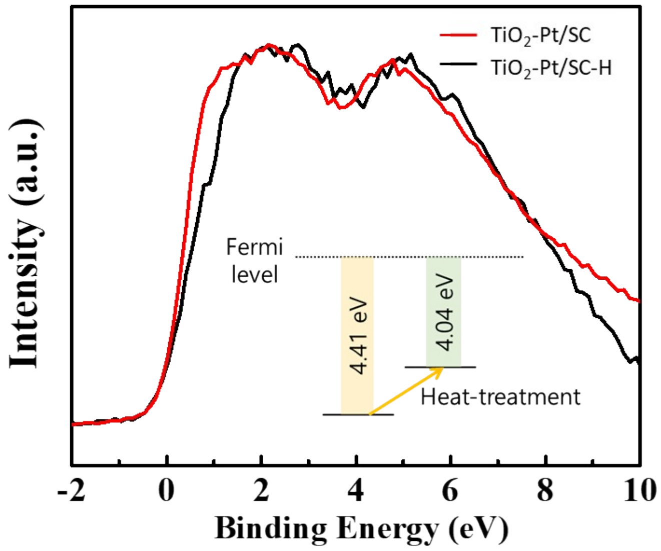

Figure S4. XPS profiles in the valence band region for TiO_2-Pt/SC and TiO_2-Pt/SC-H. The d-band center is calculated by below equation:[1]

$$d - bnad\ center\ =\ -\int_{0eV}^{10eV} [Binding\ energy\ (E) \times Intensity\ (E)]dE / \int_{0eV}^{10eV} Intensity\ (E)dE$$

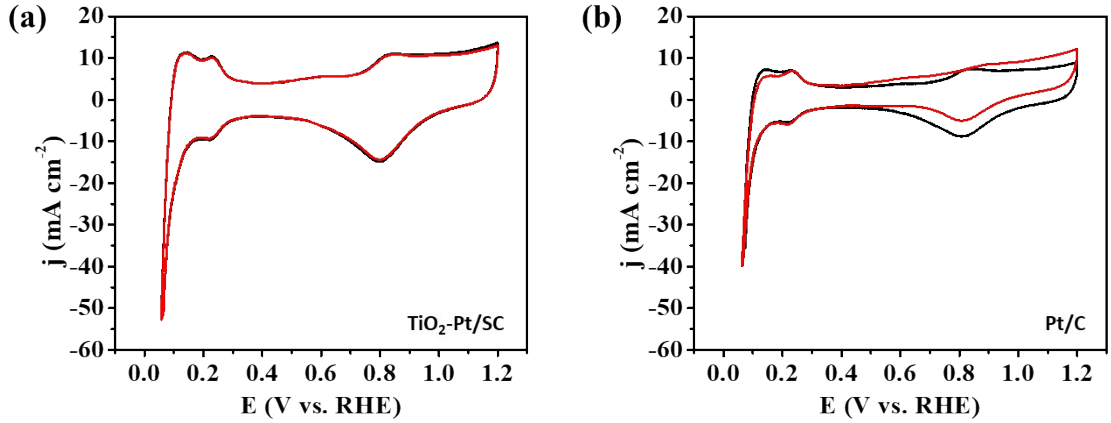

Figure S5. CV curves of MEAs before and after ADT at 70 °C of TiO_2-Pt/SC(a) and commercial Pt/C(b).

Table S1. Comparison of electrocatalytic activities of various catalysts for ORR in acid media

Catalysts	Electrolyte condition	Activity (mA cm^{-2}) 0.9 V	Scan rate	Ref.
TiO_2–Pt/SC	0.1 M $HClO_4$	**1.64**	10 mV s^{-1}	**This work**
Pt SACs	0.1 M $HClO_4$	0.51	10 mV s^{-1}	[1]
Pt/MU-MWCNT	0.1 M $HClO_4$	0.168	10 mV s^{-1}	[2]
Pt/SnO_2/C	0.1 M $HClO_4$	1.12	10 mV s^{-1}	[3]
Pt/$Ti_{0.9}Ni_{0.1}$N NTs	0.1 M $HClO_4$	1.3	10 mV s^{-1}	[4]
CrePtFe/C	0.1 M $HClO_4$	0.55	10 mV s^{-1}	[5]
CuePtTe NTs	0.1 M $HClO_4$	0.658	10 mV s^{-1}	[6]
Pd@Pt/NPs	0.1 M $HClO_4$	0.297	10 mV s^{-1}	[7]
Pt/C@NGC	0.1 M $HClO_4$	0.308	10 mV s^{-1}	[8]
PtNi-OLEA-Aged/C	0.1 M $HClO_4$	1.39	10 mV s^{-1}	[9]
Pt-Ni@PtD/G	0.1 M $HClO_4$	0.098	10 mV s^{-1}	[10]

1. Z.Song, Y. N. Zhu, H. Liu, M. N. Banis, L. Zhang, J. Li, X. Sun, Engineering the low coordinated Pt single atom to achieve the superior electrocatalytic performance toward oxygen reduction, *Small,* 2020, **16**, 2003096.

2. Q. Shu, Z. Xia, W. Wei, X. Xu, R. Sun, R. Deng, G. Sun, Controllable unzipping of carbon nanotubes as advanced Pt catalyst supports for oxygen reduction, *ACS Appl Energy Mate.,* 2019, **2**, 5446-5455.

3. K. Zhang, C. Feng, B. He, H. Dong, W. Dai, H. Lu, X. Zhang, An advanced electrocatalyst of Pt decorated SnO_2/C nanofibers for oxygen reduction reaction, *J. Electroanal. Chem.,* 2016, **781**, 198.

4. H. Nan, D. Dang, X. L. Tian, Structural engineering of robust titanium nitride as effective platinum support for the oxygen reduction reaction. *J. Mater. Chem. A,* 2018**, 6**, 6065.

5. Y. Cai, P. Gao, F. Wang, H. Zhu, Surface tuning of carbon supported chemically ordered nanoparticles for promoting their catalysis toward the oxygen reduction reaction. *Electrochi. Acta,* 2017, **246**, 671.

6. M. Dong, H. Chen, J. Xu, Z. Chen, J. Cao, Synthesis of Cu-decorated PtTe nanotubes with high electrocatalytic activity for oxygen reduction. *J. Alloys. Compd.,* 2019, **770**, 76.

7. Y. Hashiguchi, F. Watanabe, T. Honma, I. Nakamura, S. S. Poly, T. Kawaguchi, T. Fujitani, Continuous-flow synthesis of Pd@ Pt core-shell nanoparticles. *Colloids. Surf. A Physicochem. Eng. Asp.,* 2021, **620**, 126607.

8. Y. Nie, J. Deng, S. Chen, Z. Wei, Promoting stability and activity of PtNi/C for oxygen reduction reaction via polyaniline-confined space annealing strategy. *International Journal of Hydrogen Energy* 2019, **44**, 5921.

9. G. M. Leteba, Y. C. Wang, T. J. Slater, R. Cai, C. Byrne, C. P. Race, C. I. Lang, Oleylamine aging of PtNi nanoparticles giving enhanced functionality for the oxygen reduction reaction. *Nano Lett.,* 2021, **21**, 3989.

10. X. Lyu, Y. Jia, X. Mao, D. Li, G. Li, L. Zhuang, X. Yao, Gradient-concentration design of stable core–shell nanostructure for acidic oxygen reduction electrocatalysis. *Adv. Mater.*, 2020, **32**, 2003493.

Table 2.

<table>
<tr><th>Sample</th><th>OCV (V)</th><th>MPD (W/cm^2)</th><th>Current density (A/cm^2) at 0.8 V</th><th>Power density (W/cm^2) at 0.8 V</th><th>Current density (A/cm^2) at 0.6 V</th><th>Power density (W/cm^2) at 0.6 V</th><th>ECSA Loss (1.23 V, 1 h)</th><th>MPD Loss (1.23 V,1 h)</th></tr>
<tr><td>initial</td><td>1.0</td><td>1.076</td><td>0.279</td><td>0.222</td><td>1.519</td><td>0.913</td><td rowspan="2">6.41 %</td><td rowspan="2">2.42 %</td></tr>
<tr><td>After</td><td>0.978</td><td>1.05</td><td>0.199</td><td>0.159</td><td>1.359</td><td>0.818</td></tr>
<tr><td>initial</td><td>0.95</td><td>1.009</td><td>0.282</td><td>0.227</td><td>1.522</td><td>0.931</td><td rowspan="2">29.86 %</td><td rowspan="2">10.31 %</td></tr>
<tr><td>After</td><td>0.926</td><td>0.905</td><td>0.242</td><td>0.193</td><td>1.362</td><td>0.817</td></tr>
</table>

Reference

[1] G.V. Ramesh, R. Kodiyath, T. Tanabe, M. Manikandan, T. Fujita, F. Matsumoto, S. Ishihara, S. Ueda, Y. Yamashita, K. Ariga, $NbPt_3$ intermetallic nanoparticles: highly stable and CO-tolerant electrocatalyst for fuel oxidation, *ChemElectroChem* **2014**, 1, 728-732.
